# Characteristics of women with ischemic sudden cardiac death

**DOI:** 10.1080/07853890.2023.2258911

**Published:** 2023-10-05

**Authors:** I. Hookana, L. Holmström, M. A. E. Eskuri, L. Pakanen, M. M. Ollila, A. M. Kiviniemi, T. Kenttä, J. Vähätalo, M. Tulppo, E. S. Lepojärvi, T. Piltonen, J. Perkiömäki, J. T. Tikkanen, H. V. Huikuri, M. J. Junttila

**Affiliations:** a Research Unit of Biomedicine and Internal Medicine, Medical Research Center Oulu and Biocenter Oulu, University of Oulu and Oulu University Hospital, Oulu, Finland; b Forensic Medicine Unit, Finnish Institute for Health and Welfare, Oulu, Finland; c Department of Forensic Medicine, Research Unit of Internal Medicine, Medical Research Center Oulu, University of Oulu, Oulu, Finland; d Department of Obstetrics and Gynecology, Research Unit of Clinical Medicine, Medical Research Center Oulu, University of Oulu and Oulu University Hospital, Oulu, Finland

**Keywords:** Sudden cardiac death, sex, women, coronary artery disease, autopsy

## Abstract

**Background:**

Sudden cardiac death (SCD) is a significant mode of death causing 15-20% of all deaths in high-income countries. Coronary artery disease (CAD) is the most common cause of SCD in both sexes, and SCD is often the first manifestation of underlying CAD in women. This case-control study aimed to determine the factors associated with SCD due to CAD in women.

**Methods:**

The study group consisted of women with CAD-related SCD (*N* = 888) derived from the Fingesture study conducted in Northern Finland from 1998 to 2017. All SCDs underwent medicolegal autopsy. The control group consisted of women with angiographically verified CAD without SCD occurring during the 5-year-follow-up (*N* = 610). To compare these groups, we used medical records, autopsy findings, echocardiograms, and electrocardiograms (ECGs).

**Results:**

Subjects with SCD were older (73.2 ± 11.3 vs. 68.8 ± 8.0, *p* < 0.001) and were more likely to be smokers or ex-smokers (37.1% vs. 27.6%, *p* = 0.045) compared to control patients. The proportion of subjects with prior myocardial infarction (MI) was higher in controls (46.9% vs. 41.4% in SCD subjects, *p* = 0.037), but in contrast, SCD subjects were more likely to have underlying silent MI (25.6% vs. 2.4% in CAD controls, *p* < 0.001). Left ventricular hypertrophy (LVH) was more common finding in SCD subjects (70.9% vs. 55.1% in controls, *p* < 0.001). Various electrocardiographic abnormalities were more common in subjects with SCD, including higher heart rate, atrial fibrillation, prolonged QTc interval, wide or fragmented QRS complex and early repolarization. The prevalence of Q waves and T inversions did not differ between the groups.

**Conclusions:**

Underlying LVH and previous MI with myocardial scarring are common and often undiagnosed in women with CAD-related SCD. These results suggest that untreated CAD with concomitant myocardial disease is an important factor in SCD in women.

## Key Messages

- Underlying LVH and previous MI with myocardial scarring are common and often undiagnosed in women with ischemic SCD.
- Untreated CAD with concomitant myocardial disease is an important factor in SCD among women.
- Improvements in the diagnosis and management of ischemic cardiomyopathy are likely to reduce the SCD burden in women.

## 1. Introduction

Sudden cardiac death (SCD) is a major public health burden accounting for 15–20% of all deaths in high-income countries [1]. Sudden cessation of cardiac activity results from fatal arrhythmia, which is often due to structural heart disease, particularly coronary artery disease (CAD) [2]. The burden of premature death is higher for SCD than for any cancer [3]. Tools for SCD prediction and prevention are required.

Approximately one-third of all SCDs occur in women [4]. Although the incidence of SCD is significantly lower in women than in men, it still causes a remarkable health problem with 120,000 SCDs in women annually in the United States [4–6]. Many female SCD victims lack traditional SCD risk factors, such as having severely reduced left ventricular ejection fraction (LVEF) or having an established CAD diagnosis before SCD, which causes challenges in the prevention and prediction of SCD [7–10]. Coronary artery disease has also been identified as the most common cause of SCD in women [10]. In most SCD studies, the majority of SCD subjects are men and therefore, most recognized risk factors are biased towards men [10]. In addition, the decline in mortality due to SCD has been slower in women than in men [4], which indicates that more sex-specific risk stratification is needed.

Historical underrepresentation of women in CAD clinical trials has limited our understanding of CAD in women [11]. Women present more often with atypical CAD symptoms, and microvascular dysfunction is also more common among them [11–14]. Consequently, diagnostic testing may not have the same sensitivity and specificity in women as in men [15]. These disparities may contribute to a higher incidence of SCD being the initial CAD event in women compared to men [10]. However, characteristics of CAD-related SCD among women are not well investigated.

To our knowledge, no studies have compared women with CAD-related SCD to alive women with CAD. We conducted a case-control study to investigate the characteristics of CAD-related SCD among women.

## 2. Methods

### 2.1. Study population

The Fingesture (Finnish Genetic Study of Arrhythmic Events) population consisted of 5869 subjects with SCD (78.9% male; 21.1% female) in Northern Finland from 1998 to 2017. The causes of death were determined in medico-legal autopsies, which were performed for each individual in this population. Autopsies were performed at the Department of Forensic Medicine, University of Oulu, Oulu, Finland and at the Forensic Medicine Unit, Finnish Institute for Health and Welfare, Oulu, Finland. Autopsies and cause-of-death investigations were performed by experienced forensic pathologists who each perform over 100 autopsies per year, and by using contemporary guidelines for the purpose of diagnosing the cause of death. This study complied with the principles of the Declaration of Helsinki and was approved by the Ethics Committee of the Northern Ostrobothnia Hospital District and the National Authority for Medicolegal Affairs. The Ethics Committee did not require consent from the next of kin since, according to Finnish law, medicolegal autopsy does not require consent. A more detailed study protocol has been described in previous studies of this population [10,16].

**Figure 1.**
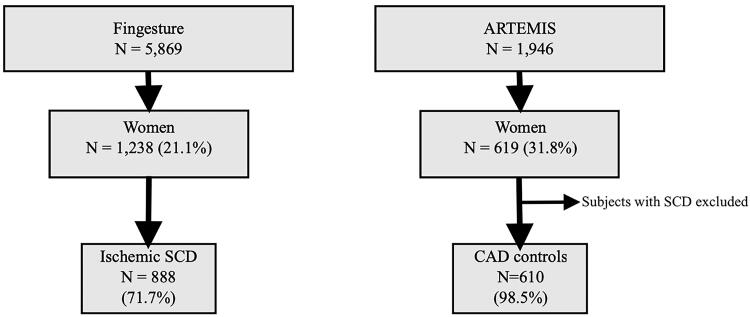
Flow chart of the study population selection. SCD indicates sudden cardiac death and CAD indicates coronary artery disease.

From this population, women with CAD-related SCD were selected as a study group, with a total of 888 subjects (Figure 1). SCD was classified as ischemic if there were culprit lesion findings of acute thrombus in coronary artery, plaque rupture or erosion, or critical coronary luminal stenosis (>75%) in a main coronary artery. The cause of SCD was also considered ischemic if the stenosis ranged from 50% to 75% and there was concomitant CAD-related myocardial disease (e.g. infarction scar) and no other cause of sudden death (e.g. cardiomyopathy, valve disease, aortic rupture, pulmonary embolism, stroke, intoxication). Individuals with non-atheromatous non-thrombotic CAD as well as cases with idiopathic infarction were not included in our study cohort.

The standard autopsy process included myocardial dissection, valve inspection, heart weight measurement, and assessment of myocardial fibrosis. This evaluation involved both macroscopic assessments as well as 3–5 histological samples from the myocardium in each subject. Myocardial fibrosis was categorized into four degrees: absence, scattered mild, patchy moderate, and substantial fibrosis. The presence of old myocardial infarction (MI) scar was also verified by histological examination of myocardial samples. An old myocardial scar was considered a manifestation of prior MI, and if there was no prior CAD diagnosis in the clinical records, it was considered as a silent MI [17].

To assess left ventricular hypertrophy (LVH), data were extracted from autopsy reports, and heart weight was classified as normal or enlarged, with a threshold of 350 grams. This threshold was selected based on autopsy findings within the Finnish population who had autopsy-verified non-cardiac causes of death [18], and the threshold aligns with the approach used for reference value in the control group, which was also derived from the general population with presumably no cardiac diseases [19]. In the present study, our focus was to evaluate the prevalence of LVH rather than the prevalence of hypertrophic cardiomyopathy, which is also associated with SCD but has a higher threshold than LVH. In addition, our aim was to assess the contributing factors for SCD rather than the primary cause of death.

We also collected data regarding smoking, which was inquired from next of kin, although the questionnaires to closest relatives were collected only from 1998 to 2007, leading to a smaller number of subjects in this subgroup.

### 2.2. Control group

Innovation to Reduce Cardiovascular Complications of Diabetes at the Intersection (ARTEMIS) was used as a source for the control group. ARTEMIS was designed to study cardiac mortality in CAD patients without diabetes compared to CAD subjects with type 2 diabetes. The ARTEMIS population consisted of 1946 patients with CAD (women 31.8%; *N* = 619). Each patient had angiographically diagnosed stenosis >50% in at least one major coronary artery. Patients who fulfilled the criteria for prophylactic implantable cardioverter defibrillator (ICD) were excluded from the study. Subjects who died from SCD during the 5-year follow up were excluded, leaving us with an eventual control group of 610 women (Figure 1).

Clinicians collected a variety of clinical data, such as smoking, medication, prior MIs, several risk markers for CAD, and adverse cardiovascular events occurring during follow-up. Data on smoking history were obtained directly from patients. To determine the prevalence of prior MIs, we utilized medical records. If no prior MIs were reported, we used pathological Q waves in two contiguous ECG leads and/or myocardial scar-related akinesia on echocardiogram as signs of silent MI. Echocardiography was performed for each patient by a cardiologist. From the echocardiography reports in the control group, the left ventricular mass was calculated using the left ventricular mass (LVM) formula (LV Mass = (0.8 × [1.04 ×((LVEDD + IVSd + PWd)³ – LVEDD³)]) + 0.6)) and considered LVH if the total exceeded the threshold of 163 g [19]. Informed consent was obtained from all the participants. The study was conducted in accordance with the principles of the Declaration of Helsinki and the study protocol was approved by the ethical committee of the Northern Ostrobothnia Hospital District. A more detailed research design for the ARTEMIS study was described in the study by Junttila et al. [20].

### 2.3. ECG analysis

ECG was available for 171 (19.3%) subjects in the SCD group and for 608 (99.7%) subjects in the control group. In the SCD group, antemortem recorded ECGs, if available, were independently analyzed by two investigators. The median time between the latest antemortem ECG and SCD was 1 year (interquartile range, 0.0, 4.0 years). In the control group, ECGs were recorded 3-6 months after coronary angiography and analyzed using custom-made software.

In addition to measuring standard intervals such as PR, QRS, QTc and JTc, we analyzed T wave inversions, bundle branch blocks (BBBs), QRS fragmentations (fQRS), Q waves and early repolarization (ER). Data on atrial fibrillation (AF) and atrial flutter (AFL) was also collected. When assessing QRS duration, we excluded patients with bundle branch block. We classified a QRS duration ≥110 ms as prolonged. The PR interval was classified as shortened if <120 ms and prolonged if >200 ms; when assessing the PR interval, subjects with AF or AFL were excluded. QTc duration ≥460 ms was considered prolonged. Additionally, we compared the JTc intervals between groups to minimize the distorting effect of the widened QRS. All interval measurements were evaluated using the mean intervals of all ECG leads.

T wave inversions were classified by coronary regions (lateral: I, aVL, V4-V6, anterior: V1-V3, inferior: II, III, aVF) and the regional T inversion criteria were T inversions (>0.1 mV) in at least two contiguous leads in the same region. Regional classification was also used when assessing the fQRS, ER, and Q waves. fQRS was defined as at least two additional spikes in QRS complex that could not be regarded as ER whereas at least two slurred or notched J point elevations ≥0.1 mV was characteristic of ER. BBBs were excluded when assessing the ER and fQRS. The Q waves were assessed using the standard Minnesota Criteria. To determine left ventricular hypertrophy, we used the Cornell criteria (S wave in V3 and R wave in aVL > 20 mm) and the Sokolow-Lyon criteria (tallest R wave in V5 or V6 and S wave in V1 > 35 mm).

### 2.4. Statistical analysis

Statistical analyses were performed using the Statistical Package for Social Studies 21.0 (SPSS Inc. Chicago, IL). When comparing continuous variables, we used Student’s t-test considering the distribution of the data as Gaussian if the skewness was within ±1. Pearson’s Chi-Square test, followed by a post-hoc test (Bonferroni), was used to compare categorical variables. Statistical significance was defined as a two two-sided p-value of <0.05.

## 3. Results

### 3.1. Patient history characteristics

The characteristics of the CAD-related SCD subjects and CAD control patients are shown in Table 1. The study group consisted of 888 women with CAD-related SCD, of whom 661 had not been previously diagnosed with CAD. The control group consisted of 610 women with angiographically diagnosed CAD without SCD during the 5-year-follow-up. Women in SCD group were older compared to women in control group (73.2 ± 11.3 years vs. 68.8 ± 8.0 years, *p* < 0.001). Women with SCD had lower BMI (27.4 ± 6.4 kg/m^2^ vs. 28.6 ± 5.0 kg/m^2^, *p* < 0.001) as well as lower prevalence of obesity (BMI > 30 kg/m^2^) than women in control group (29.0% vs. 37.0%, *p* = 0.002). Additionally, underweight (BMI < 18.5 kg/m^2^) was more common finding in women with CAD-related SCD (5.5% vs. 0.3% in controls, *p* < 0.001).

**Table 1.** Characteristics of CAD-related SCD victims and CAD control patients.

	SCDs (*N* = 888)	Controls (*N* = 610)	*p*-value
Age (y)*	73.2 ± 11.3	68.8 ± 8.0	*p* < 0.001
BMI (kg/m²)*	27.4 ± 6.4	28.6 ± 5.0	*p* < 0.001
BMI < 18.5* (%, n)BMI > 30* (%, n)	5.5 (39/703)29.0 (204/703)	0.3 (2/610)37.0 (226/610)	*p* < 0.001*p* = 0.002
Current smoker* (%, n)Prior smoker* (%, n)Current or prior smoker* (%, n)	30.2 (35/116)6.9 (8/116)37.1 (43/116)	6.3 (38/608)21.4 (130/608)27.6 (168/608)	*p* < 0.001*p* < 0.001*p* = 0.045

All the subjects were females. Continuous variables are expressed as the mean ± SD. SCD: sudden cardiac death; CAD: coronary artery disease; BMI: body mass index. *Significant difference between groups (*p* < 0.05).

Smoking data were available for 116 subjects (13.1%) in the SCD group and 608 subjects (99.7%) in the control group. Almost a third of subjects with SCD (30.2%) were reported to be active smokers whereas in control group, the corresponding percentage was significantly lower; 6.3% (*p* < 0.001). In contrast, women in control group had quit smoking more often compared to SCD group (21.4% vs. 6.9%, *p* < 0.001). Overall, the prevalence of current or prior smoking was higher in subjects with SCD (37.1% vs. 27.6% in controls, *p* = 0.045).

### 3.2. Prior MIs

**Figure 2.**
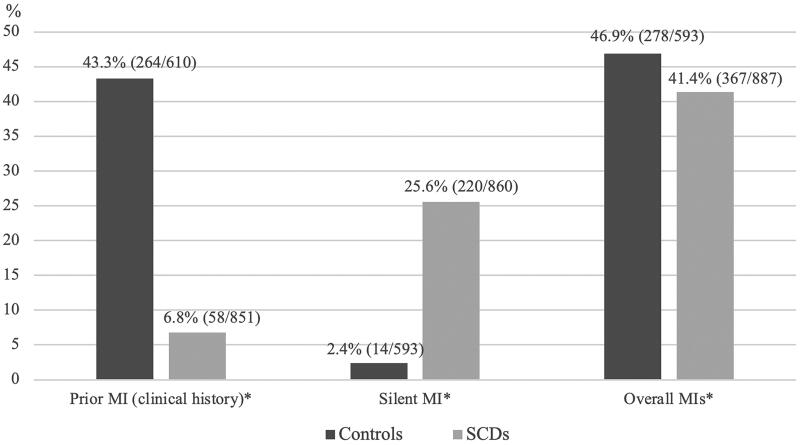
Prior MIs among CAD-related SCD subjects and CAD control patients. All the subjects were females. SCD, sudden cardiac death; CAD, coronary artery disease; MI, myocardial infarction; Prior MI, clinical history of MI; Silent MI, findings related to infarction scar with no MI/CAD history. Overall MIs include reported MIs, silent MIs and infarction scars discovered at the autopsy. *Significant difference (p < 0.05) between groups.

Clinical history of MI was higher in control group compared to SCD group: 43.3% vs. 6.8% (*p* < 0.001; Figure 2). In contrast, in subjects with SCD, the proportion of silent MIs was substantially higher compared to that in subjects in control group as over one quarter of SCD victims had had silent MI (25.6% vs. 2.4%, *p* < 0.001; Figure 2). Overall, prevalence of MI remained slightly higher in control group compared to SCD group (46.9%vs. 41.4%, *p* = 0.037; Figure 2). Silent MIs accounted for 59.9% of all MIs in SCD cases.

### 3.3. Left ventricular hypertrophy

LVH, assessed either *via* heart weight at autopsy or echocardiography in control subjects, was more frequent finding among subjects with SCD (70.9% vs. 55.1% in controls, *p* < 0.001; Table 2). Majority of the SCD subjects (80.0%) had either LVH or prior MI whereas in controls, the corresponding proportion was 73.5% (*p* = 0.004, Table 2).

**Table 2.** Prevalence of LVH among subjects with CAD-related SCD and CAD control patients.

	SCDs (*N* = 887)	Controls (*N* = 610)	*p*-value
Heart weight (g)	404.1 ± 95.6		
LVM (g)		164.5 (142.5, 199.4)	
LVH (%, *n*) LVH or prior MI (%, *n*)	70.9 (629/887)80.0 (710/887)	55.1 (336/610)73.5 (443/603)	*p* < 0.001,*p* = 0.004

All the subjects were females. Continuous variables are expressed as mean *±*SD or, if not normally distributed, as median (interquartile range). SCD, sudden cardiac death; CAD, coronary artery disease; LVM, left ventricular mass; LVH, left ventricular hypertrophy; MI, myocardial infarction. *=Significant difference between groups (*p* < 0.05).

### 3.4. Other autopsy findings in SCD subjects

In the macroscopic evaluation, coronary artery stenosis of 75% to 90% occurred in 44.3% of SCD subjects, while complete occlusion was found in 29.8%. Stenosis of 50% to 75% was noted in 23.9%, and no significant coronary artery stenosis was detected in 2.0% of SCD cases.

In the SCD group, myocardial fibrosis was histologically evaluated in each subject. The prevalence of fibrosis was as follows: scattered mild fibrosis was present in 28.0% of subjects, patchy moderate fibrosis in 52.2%, substantial fibrosis in 9.5%, and 10.4% of SCD subjects did not exhibit any grade of myocardial fibrosis.

### 3.5. ECG findings

The mean heart rate on ECG recordings was 76 beats/min in the SCD group and 67 beats/min in the control group (*p* < 0.001). The prevalence of LVH as an ECG finding did not differ between the groups when using Cornell’s criteria or the Sokolow-Lyon criteria (Table 3). Women in SCD group had longer QTc interval than control subjects (437.3 ms ± 7.3 ms vs. 429.0 ms ± 24.3 ms, *p* = 0.006) and JTc interval (342.0 ms ± 35.7 ms vs. 335.0 ms ± 21.8 ms in CAD controls, *p* = 0.036). Approximately one in four subjects had prolonged QTc duration ≥460 ms in SCD group and only one in ten CAD patient control had prolonged QTc (25.7% vs. 11.1%, *p* < 0.001). After excluding subjects with QRS duration ≥110 ms, the prevalence of prolonged QTc duration remained higher in SCD group (18.2% vs. 7.0% in CAD controls, *p* < 0.001). The difference in JTc interval was smaller but significant between the groups (Table 3). Additionally, prolonged QRS duration ≥110 ms was three times more common finding in the SCD group compared to the control group (9.5% vs. 2.7%, *p* < 0.001).

**Table 3.** Prevalence of electrocardiographic risk markers among CAD-related SCD subjects and CAD control patients.

	SCDs (*N* = 171)	Controls (*N* = 595)	*p*-value
QTc (ms)*	437.3 ± 37.3	429.0 ± 24.3	0.006
JTc (ms)*	342.0 ± 35.7	335.9 ± 21.8	0.036
PR (ms)*	167.5 ± 32.5	173.0 ± 29.2	0.048
Heart rate (bpm)*	76.3 ± 15.7	67.3 ± 8.61	<0.001
LVH (Sokolow-Lyon) (%, *n*)	4.1 (7/171)	4.7 (28/595)	0.838
LVH (Cornell) (%, *n*)	18.7 (32/171)	18.0 (107/595)	0.910
QTc ≥ 460 ms* (%, *n*)	25.7 (44/171)	11.1 (66/595)	<0.001
QTc ≥ 460 ms* (%, *n*) *Subjects with QRS ≥ 110 ms excluded*	18.2 (26/143)	7.0 (38/544)	<0.001
QRS ≥ 110 ms* (%, *n*)	9.5 (15/158)	2.7 (15/555)	<0.001
PR < 120 ms (%, *n*)	2.8 (4/145)	1.6 (9/577)	0.483
PR > 200 ms (%, *n)*	14.5 (21/145)	14.9 (86/577)	1.000
Atrial fibrillation* (%, *n*)	14.6 (25/171)	3.2 (19/598)	<0.001
ER* (%, *n*) Lateral Anterior Inferior	24.1 (38/158)14.6 (23/158)0.6 (1/158)9.5 (15/158)	16.6 (92/554)9.6 (53/554)0 (0/554)7.6 (42/554)	0.0360.0800.2220.506
Q waves (%, *n*) Lateral Anterior Inferior	9.4 (16/171)0.6 (1/171)2.3 (4/171)6.4 (11/171)	6.2 (37/595)0.3 (2/595)1.0 (6/595)5.2 (31/595)	0.1711.0000.2430.568
T inversions (%, *n*) Lateral Anterior* Inferior	19.3 (33/171)13.5 (23/171)5.3 (9/171)6.4 (11/171)	23.9 (142/594)13.8 (82/594)11.1 (66/594)5.2 (31/594)	0.2171.0000.0280.568
fQRS* (%, *n*) Lateral* Anterior* Inferior*	42.4 (67/158)14.6 (23/158)10.8 (17/158)36.1 (57/158)	23.8 (132/554)2.3 (12/554)3.2 (18/554)20.0 (111/554)	<0.001< 0.001< 0.001< 0.001

All the subjects were females. Continuous variables are expressed as mean *±*SD. SCD, sudden cardiac death; CAD, coronary artery disease; LVH, left ventricular hypertrophy; ER, early repolarization; fQRS, fragmented QRS. When comparing the prevalence of prolonged QRS, ER and fQRS, subjects with bundle branch block were excluded. When assessing PR interval, subjects with atrial fibrillation or atrial flutter were excluded. *Significant difference between groups (*p* < 0.05).

The prevalence of AF was higher in SCD group than in control group (14.6% vs. 3.2%, *p* < 0.001). ER was found in 24.1% of SCD patients and in 16.6% of control subjects (*p* = 0.036). Also, fQRS was more frequent finding in SCD subjects (42.4% vs. 23.8% in controls, *p* < 0.001). T wave inversions in anterior ECG leads were observed more often in control group than in SCD group (11.1% vs. 5.3%, *p* = 0.028) but the total prevalence of T wave inversions did not differ between groups. The prevalence of pathological Q waves did not differ between the groups. All the ECG analyses are presented in Table 3.

### 3.6. ECG findings in SCD subjects with left ventricular hypertrophy

Among the 629 SCD subjects with LVH, prior ECG recordings were available for 18.8% (*N* = 118). Only 24.6% of SCD subjects with LVH fulfilled Cornell’s criteria. The Sokolow-Lyon criteria were less sensitive in detecting LVH, as only 4.2% of SCD subjects with LVH met these criteria. The prevalence of T wave inversions was 23.7%. Prolonged QRS without BBB was found in 12.1% of the subjects. The prevalence of BBB was 9.3%. Among SCD subjects with LVH, 48.3% had at least one ECG abnormality (Cornell > 20 mm, Sokolow-Lyon > 35 mm, T wave inversions, QRS ≥ 110 ms, BBB). All the ECG findings of SCD subjects with LVH are presented in Table 4.

**Table 4.** Prevalence of electrocardiographic risk markers among CAD-related SCD subjects with left ventricular hypertrophy.

Any ECG abnormality (%, n)	48.3 (57/118)
LVH (Cornell) (%, n)	24.6 (29/118)
LVH (Sokolow-Lyon) (%, n)	4.2 (5/118)
LVH (Cornell or Sokolow-Lyon) (%, n)	28.0 (33/118)
T wave inversions (%, n)	23.7 (28/118)
QRS ≥ 110 ms (%, n) *BBBs excluded*	12.1 (13/107)
BBB (LBBB or RBBB) (%, n)	9.3 (11/118)

ECG was available for 118 sudden cardiac death (SCD) subjects with LVH. CAD, coronary artery disease; LVH, left ventricular hypertrophy; BBB, bundle branch block. “Any ECG abnormality” includes ECG-LVH (Cornell or Sokolow-Lyon), T wave inversions, BBB or QRS ≥ 110 ms.

### 3.7. ECG findings in SCD subjects with silent myocardial infarction

Of the SCD subjects with silent MI (*N* = 220), ECG-recording was available for 17.7% (*N* = 39). The prevalence of pathological Q waves in this group was 3/39 and fQRS was detected in 15 of 36 subjects. The prevalence of QRS prolongation without a BBB was 2/36. Inverted T waves were found in six of the 39 subjects. Overall, 56.4% of subjects with silent MI had at least one of the ECG abnormalities mentioned above (QRS ≥ 110 ms, BBB, T inversions, Q waves, fQRS). All the ECG findings of SCD victims with a silent MI are presented in Table 5. Comparison of left ventricular ejection fraction between SCD cases and controls is provided in the Supplemental Figure.

**Table 5.** Prevalence of electrocardiographic risk markers among CAD-related SCD subjects with silent myocardial infarction.

Any ECG abnormality (%, n)	56.4 (22/39)
Q waves (%, n)	7.7 (3/39)
Fragmented QRS (%, n) *BBBs excluded*	41.7 (15/36)
T wave inversions (%, n)	15.4 (6/39)
QRS ≥ 110 ms (%, n) *BBBs excluded*	5.6 (2/36)
BBB (LBBB or RBBB) (%, n)	7.6 (3/39)

ECG was available for 39 sudden cardiac death (SCD) subjects with silent myocardial infarction. CAD indicates coronary artery disease and BBB indicates bundle branch block. “Any ECG abnormality” includes Q waves, fragmented QRS, T wave inversions, QRS ≥ 110 ms or BBB.

## 4. Discussion

In this case-control study, we compared the characteristics of women with CAD-related SCD with female CAD patients without SCD. In most subjects in the SCD group, sudden cardiac death was the first manifestation of underlying CAD. The number of subjects with a clinical history of MI was lower in SCD subjects than in controls; in contrast, the proportion of silent MIs was substantially higher in subjects with SCD. Left ventricular hypertrophy was a more frequent finding in the SCD group. Various ECG abnormalities were more common in SCD subjects, including higher heart rate, atrial fibrillation, prolonged QTc interval (also after excluding subjects with QRS ≥ 110 ms), wide or fragmented QRS complex and early repolarization. The prevalence of Q waves and T inversions did not differ between the groups.

In Europe, CAD accounts for approximately 23% of all deaths in women [20]. Historically, the underrepresentation of women in the CAD-focused clinical trials has led to an incomplete understanding of sex-specific factors in CAD, consequently biasing clinical recommendations towards men [11]. This imbalance presents challenges in accurately diagnosing and treating CAD in women. Previous studies have demonstrated that the risk factors for CAD and their relative importance differ between women and men [12,14]. Current literature also indicates that women are more likely to experience atypical angina symptoms and have a higher prevalence of microvascular dysfunction compared to men [11–13]. Consequently, women are less frequently referred for diagnostic procedures compared to men, and the diagnostic accuracy of the ECG stress test is lower in women than in men [15,21,22]. Treatment guidelines primarily based on evidence from male-dominated trials may also contribute to suboptimal care for women. This is evident in the lower referral rates for appropriate therapeutic procedures, fewer guideline-recommended medical therapies for secondary prevention, and higher complication rates after acute MI in women compared to men [11,12,21,22,23]. Given these factors, there is a need for more comprehensive study data on women with CAD to develop sex-specific recommendations and improve the management of CAD in women.

In this study, the proportion of unrecognized MIs among female SCD subjects was high: two thirds of all myocardial scars were manifestations of silent MIs among SCD subjects. Data from other studies showing evidence of silent MI as a risk factor for SCD support these findings [17,24,25]. Myocardial scarring provides a substrate for fatal arrhythmia and therefore previous MI increases the risk of SCD [17,24]. Myocardial infarctions are generally more often unrecognized in the female population [13,25–27]. This might be due to several reasons, such as insufficient awareness of sex-specific CAD risk factors and presentation of the disease among female patients, as well as among physicians [11–13]. Often, atypical angina pectoris is mistakenly interpreted as extracardiac symptoms in women [11–13]. In addition, medical training focuses on the recognition of male pattern symptoms, probably because of the high prevalence of CAD in men [11,13]. If MI remains unrecognized, patients do not receive appropriate treatment and secondary preventive therapies, and are therefore more vulnerable to developing new events, such as ischemic heart failure (HF) and/or severe arrhythmia leading to SCD. These findings indicate the urgent need for more sex-specific clinical recommendations to prevent and detect MIs more efficiently in the female population.

In the present study, the proportion of subjects with LVH in SCD group was high with a total of 70.9%, which was higher number compared to the alive CAD patients (55.1%). Previous studies have shown that LVH is a risk factor for SCD, particularly in the presence of myocardial scarring, ischemia, or fibrosis [2,28–31]. In a study by Kaikkonen et al. [30], the incidence of LVH was higher among CAD-related SCD victims than among acute MI survivors, even though there was no difference in the history of hypertension between the groups, suggesting that in addition to hypertension, other factors causing cardiac hypertrophy may predispose to SCD. In studies based on the Framingham study population [6,31], LVH appeared to be a stronger predictor of SCD in males than in females. However, population-based studies may be underpowered owing to the low number of women with SCD. A post-hoc analysis of the LIFE study [32] showed that regression of ECG-LVH during antihypertensive therapy is associated with reductions in the risk of SCD independently of blood pressure lowering and other known risk factors of SCD. Considering the findings of the LIFE study [32] and the significant role of hypertension as a risk factor for SCD [32–34], clinicians should focus on efficient antihypertensive therapy also in women to lower the risk of SCD.

Current cigarette smoking is strongly associated with SCD risk [4,6,35]. In the Nurses’ Health Study [35], among women with and without CAD, current smokers had a significantly higher risk of SCD than women who had never smoked, and the risk of SCD declined after smoking cessation. In our study, nearly one third of CAD-related SCD victims were current smokers, whereas in control subjects, the corresponding percentage was 6.3%. Unlike SCD subjects, control patients were more likely to have quit smoking which might be due to the awareness of their heart disease and clinical guidance provided by healthcare professionals.

Obesity is associated with an increased risk of SCD [1,4]. However, in our study, obesity was observed more frequently in the control group than in the SCD group. This might result from the high prevalence of type 2 diabetes (41.4%) in the control group. The prevalence of underweight was higher in SCD victims, which might be related to the higher rates of smoking in the SCD group.

Abnormalities in ECG may raise the suspicion of an underlying heart disease associated with SCD. In this study, when we assessed ECG abnormalities only in SCD subjects with left ventricular hypertrophy or silent MI, approximately half of the subjects had at least one ECG abnormality related to the underlying heart disease. When comparing the prevalence of Q waves and T inversions among all subjects, we did not find significant differences between female SCD victims and female CAD patients. Additionally, there were no significant differences when comparing subjects with ECG-LVH, which might have resulted from the high rates of LVH in both groups. However, a fragmented QRS-complex, a possible sign of myocardial scarring [36], was detected in nearly half of the subjects with SCD, which is concordant with the findings of other studies associating fragmented QRS with the occurrence of SCD [37,38]. In our study, the prevalence of prolonged QTc was higher in subjects with SCD, which is consistent with prior studies that defined QTc prolongation as a predictor of SCD [39,40]. The role of prolonged QRS duration as a risk factor for SCD has been well-established [40,41], and our study results support this finding, as prolonged QRS was more frequent finding in SCD subjects. In previous studies [42], atrial fibrillation independently increased the risk of SCD, which is in line with our study findings, with a higher prevalence of atrial fibrillation among SCD subjects.

## 5. Limitations

Our study has some limitations that need to be addressed. Antemortem ECGs and echocardiograms were available for minority of SCD subjects whereas in alive CAD controls, these were routine examinations that were performed for each patient in the cohort. Moreover, the timeframe between the latest ECG and SCD varied among the SCD subjects. Also, subjects with LVEF <35% were excluded from Artemis study from where the control population was drawn. Most of the SCD cases had a limited amount of pre-SCD healthcare contacts and medical records, and hence we were able to gather reliable data on medications only for subjects in the control group. A major strength of our study is the large, unselected sample of CAD-SCD in women at the community-level, but slightly higher mean age in comparison to controls may result in a limitation.

In the control group, silent MI was diagnosed using ECG changes and echocardiography, while in the SCD group, it was detected through autopsy. Similarly, LVH prevalence was compared using echocardiography for control subjects and heart weight for SCD subjects. Additionally, in the control group, CAD was defined as a stenosis >50% in a major coronary artery while in the SCD group, CAD was primarily defined as a stenosis exceeding 75% in a major epicardial coronary artery. However, cases with a 50–75% stenosis were also classified as ischemic if there was concomitant CAD-related myocardial disease (e.g. scar). These differences in diagnostic methods and diagnostic criteria could introduce a potential limitation to our study.

However, even when these significant limitations are considered, it still seems important to show the differences between SCD victims and living CAD subjects rather than presenting the characteristics of SCD victims on their own.

## 6. Conclusions

In this study, we found that underlying LVH and previous MI with myocardial scarring are common and often undiagnosed in women with ischemic SCD. Several ECG abnormalities were more common in the subjects with SCD. These results suggest that undiagnosed and untreated CAD with concomitant MI is a significant factor related to SCD in women. Improvements in the diagnosis and management of ischemic cardiomyopathy are likely to reduce the SCD burden in women.

## Supplementary Material

Supplemental Material

## Data Availability

The data supporting the findings of this study can be obtained from the corresponding author, MJ Junttila, upon reasonable request.

## References

[1] Wong CX, Brown A, Lau DH, et al. Epidemiology of sudden cardiac death: global and regional perspectives. Heart Lung Circ. 2019;28(1):1–10. doi: 10.1016/j.hlc.2018.08.026. 30482683

[2] Myerburg RJ, Junttila MJ. Sudden cardiac death caused by coronary heart disease. Circulation. 2012;125(8):1043–1052. doi: 10.1161/CIRCULATIONAHA.111.023846. 22371442

[3] Stecker EC, Reinier K, Marijon E, et al. Public health burden of sudden cardiac death in the United States. Circ Arrhythm Electrophysiol. 2014;7(2):212–217. doi: 10.1161/CIRCEP.113.001034. 24610738 PMC4041478

[4] Albert CM, Chae CU, Grodstein F, et al. Prospective study of sudden cardiac death among women in the United States. Circulation. 2003;107(16):2096–2101. doi: 10.1161/01.CIR.0000065223.21530.11. 12695299

[5] Schatzkin A, Cupples LA, Heeren T, et al. Sudden death in the framingham heart study. Differences in incidence and risk factors by sex and coronary disease status. Am J Epidemiol. 1984;120(6):888–899. doi: 10.1093/oxfordjournals.aje.a113960. 6239541

[6] Kannel WB, Wilson PW, D’Agostino RB, et al. Sudden coronary death in women. Am Heart J. 1998;136(2):205–212. doi: 10.1053/hj.1998.v136.90226. 9704680

[7] Chugh SS, Uy-Evanado A, Teodorescu C, et al. Women have a lower prevalence of structural heart disease as a precursor to sudden cardiac arrest: the Ore-SUDS (Oregon sudden unexpected death study). J Am Coll Cardiol. 2009;54(22):2006–2011. doi: 10.1016/j.jacc.2009.07.038. 19926005 PMC2850557

[8] Reinier K, Stecker EC, Uy-Evanado A, et al. Sudden cardiac death as first manifestation of heart disease in women. Circulation. 2020;141(7):606–608. doi: 10.1161/CIRCULATIONAHA.119.044169. 32065764 PMC7122833

[9] Simmons A, Pimentel R, Lakkireddy D. Sudden cardiac death in women. Rev Cardiovasc Med. 2012;13(1):e37–e42. doi: 10.3909/ricm0589. 22565537

[10] Haukilahti MAE, Holmström L, Vähätalo J, et al. Sudden cardiac death in women. Circulation. 2019;139(8):1012–1021. doi: 10.1161/CIRCULATIONAHA.118.037702. 30779638

[11] Mehta LS, Beckie TM, DeVon HA, et al. Acute myocardial infarction in women: a scientific statement from the American heart association. Circulation. 2016;133(9):916–947. doi: 10.1161/CIR.0000000000000351. 26811316

[12] Humphries KH, Izadnegahdar M, Sedlak T, et al. Sex differences in cardiovascular disease - Impact on care and outcomes. Front Neuroendocrinol. 2017;46:46–70. doi: 10.1016/j.yfrne.2017.04.001. 28428055 PMC5506856

[13] McSweeney JC, Cody M, O’Sullivan P, et al. Women’s early warning symptoms of acute myocardial infarction. Circulation. 2003;108(21):2619–2623. doi: 10.1161/01.CIR.0000097116.29625 14597589

[14] Michos ED, Vasamreddy CR, Becker DM, et al. Women with a low framingham risk score and a family history of premature coronary heart disease have a high prevalence of subclinical coronary atherosclerosis. Am Heart J. 2005;150(6):1276–1281. doi: 10.1016/j.ahj.2005.02.037. 16338271

[15] Kwok Y, Kim C, Grady D, et al. Meta-analysis of exercise testing to detect coronary artery disease in women. Am J Cardiol. 1999;83(5):660–666. doi: 10.1016/s0002-9149(98)00963-1. 10080415

[16] Holmström L, Juntunen S, Vähätalo J, et al. Plaque histology and myocardial disease in sudden coronary death: the fingesture study. Eur Heart J. 2022;43(47):4923–4930. doi: 10.1093/eurheartj/ehac533. 36172703 PMC9748531

[17] Vähätalo JH, Huikuri HV, Holmström LTA, et al. Association of silent myocardial infarction and sudden cardiac death. JAMA Cardiol. 2019;4(8):796–802. doi: 10.1001/jamacardio.2019.2210. 31290935 PMC6624824

[18] Lehti H. Normal weights of human organs. A postmortem study on cases of death from external causes. Helsinki: Department of Forensic Medicine, University of Helsinki, 1971.

[19] Lang RM, Badano LP, Mor-Avi V, et al. Recommendations for cardiac chamber quantification by echocardiography in adults: an update from the American society of echocardiography and the european association of cardiovascular imaging. J Am Soc Echocardiogr. 2015;28(1):1–39e14. doi: 10.1016/j.echo.2014.10.003. 25559473

[20] Junttila MJ, Kiviniemi AM, Lepojärvi ES, et al. Type 2 diabetes and coronary artery disease: preserved ejection fraction and sudden cardiac death. Heart Rhythm. 2018;15(10):1450–1456. doi: 10.1016/j.hrthm.2018.06.017. 30274618

[21] Stramba-Badiale M, Fox KM, Priori SG, et al. Cardiovascular diseases in women: a statement from the policy conference of the european society of cardiology. Eur Heart J. 2006;27(8):994–1005. doi: 10.1093/eurheartj/ehi819. 16522654

[22] Arnold AL, Milner KA, Vaccarino V. Sex and race differences in electrocardiogram use (the national hospital ambulatory medical care survey). Am J Cardiol. 2001;88(9):1037–1040. doi: 10.1016/s0002-9149(01)01987-7. 11704006

[23] Lansky AJ, Hochman JS, Ward PA, et al. Percutaneous coronary intervention and adjunctive pharmacotherapy in women: a statement for healthcare professionals from the American heart association. Circulation. 2005;111(7):940–953. doi: 10.1161/01.CIR.0000155337.50423.C9. 15687113

[24] Adabag AS, Peterson G, Apple FS, et al. Etiology of sudden death in the community: results of anatomical, metabolic, and genetic evaluation. Am Heart J. 2010;159(1):33–39. Jandoi: 10.1016/j.ahj.2009.10.019. 20102864 PMC2905235

[25] Van der Ende MY, Juarez-Orozco LE, Waardenburg I, et al. Sex-Based differences in unrecognized myocardial infarction. J Am Heart Assoc. 2020;9(13):e015519. doi: 10.1161/JAHA.119.015519. 32573316 PMC7670510

[26] de Torbal A, Boersma E, Kors JA, et al. Incidence of recognized and unrexognized myocardial infarction in men and women aged 55 and older: the rotterdam study. Eur Heart J. 2006;27(6):729–736. doi: 10.1093/eurheartj/ehi707. 16478749

[27] Kannel WB. Silent myocardial ischemia and infarction: insights from the framingham study. Cardiol Clin. 1986;4(4):583–591. doi: 10.1016/S0733-8651(18)30577-0. 3779719

[28] Shenasa M, Shenasa H. Hypertension, left ventricular hypertrophy, and sudden cardiac death. Int J Cardiol. 2017;237:60–63. doi: 10.1016/j.ijcard.2017.03.002. 28285801

[29] Vähätalo JH, Holmström LTA, Pylkäs K, et al. Genetic variants associated with sudden cardiac death in victims with single vessel coronary artery disease and left ventricular hypertrophy with or without fibrosis. Front Cardiovasc Med. 2021;8:755062. doi: 10.3389/fcvm.2021.755062. 35087879 PMC8788946

[30] Kaikkonen KS, Kortelainen M-L, Huikuri HV. Comparison of risk profiles between survivors and victims of sudden cardiac death from an acute coronary event. Ann Med. 2009;41(2):120–127. doi: 10.1080/07853890802213295. 18720091

[31] Haider AW, Larson MG, Benjamin EJ, et al. Increased left ventricular mass and hypertrophy are associated with increased risk for sudden death. J Am Coll Cardiol. 1998;32(5):1454–1459. doi: 10.1016/s07351097(98)00407-0. 9809962

[32] Wachtell K, Okin PM, Olsen MH, et al. Regression of electrocardiographic left ventricular hypertrophy during antihypertensive therapy and reduction in sudden cardiac death: the LIFE study. Circulation. 2007;116(7):700–705. doi: 10.1161/CIRCULATIONAHA.106.666594. 17664372

[33] Verdecchia P, Angeli F, Cavallini C, et al. Sudden cardiac death in hypertensive patients. Hypertension. 2019;73(5):1071–1078. doi: 10.1161/HYPERTENSIONAHA.119.12684. 30827144

[34] Laukkanen JA, Jennings JR, Kauhanen J, et al. Relation of systemic blood pressure to sudden cardiac death. Am J Cardiol. 2012;110(3):378–382. doi: 10.1016/j.amjcard.2012.03.035. 22521306

[35] Sandhu RK, Jimenez MC, Chiuve SE, et al. Smoking, smoking cessation, and risk of sudden cardiac death in women. Circ Arrhythm Electrophysiol. 2012;5(6):1091–1097. doi: 10.1161/CIRCEP.112.975219. 23233741 PMC4025959

[36] Varriale P, Chryssos BE. The RSR′ complex not related to right bundle branch block: diagnostic value as a sign of myocardial infarction scar. Am Heart J. 1992;123(2):369–376. doi: 10.1016/0002-8703(92)90648-f. 1736572

[37] Rosengarten JA, Scott PA, Morgan JM. Fragmented QRS for the prediction of sudden cardiac death: a meta-analysis. Europace. 2015;17(6):969–977. doi: 10.1093/europace/euu279. 25355781

[38] Das MK, Khan B, Jacob S, et al. Significance of a fragmented QRS complex versus Q wave in patients with coronary artery disease. Circulation. 2006;113(21):2495–2501. doi: 10.1161/CIRCULATIONAHA.105.595892. 16717150

[39] Algra A, Tijssen JG, Roelandt JR, et al. QTc prolongation measured by standard 12-lead electrocardiography is an independent risk factor for sudden death due to cardiac arrest. Circulation. 1991;83(6):1888–1894. doi: 10.1161/01.cir.83.6.1888. 2040041

[40] Tikkanen JT, Kentta T, Porthan K, et al. Risk of sudden cardiac death associated with QRS, QTc, and JTc intervals in the general population. Heart Rhythm. 2022;19(8):1297–1303. doi: 10.1016/j.hrthm.2022.04.016. 35472593

[41] Kurl S, Mäkikallio TH, Rautaharju P, et al. Duration of QRS complex in resting electrocardiogram is a predictor of sudden cardiac death in men. Circulation. 2012;125(21):2588–2594. doi: 10.1161/CIRCULATIONAHA.111.025577. 22615341

[42] Chen LY, Sotoodehnia N, Bůžková P, et al. Atrial fibrillation and the risk of sudden cardiac death. JAMA Intern Med. 2013;173(1):29–35. doi: 10.1001/2013.jamainternmed.744. 23404043 PMC3578214

