# Supplementary material for: Characteristics of women with ischemic sudden cardiac death
Source: Ann Med. 2023 Oct 5;55(2):2258911. doi: 10.1080/07853890.2023.2258911 (PMC10557538; doi:10.1080/07853890.2023.2258911)
Supplement: Supplemental Material [file IANN_A_2258911_SM9872.docx]

**Supplemental Figure. Left ventricular ejection fraction (LVEF) among CAD-related SCD subjects and CAD control patients**

All the subjects were females. SCD, sudden cardiac death; CAD, coronary artery disease; LVEF, left ventricular ejection fraction. The control group, drawn from the Artemis cohort, did not include individuals with LVEF<35 %. *Significant difference between groups (p<0.05).
